# Capillary-assisted flat-field formation: a platform for advancing nanoparticle tracking analysis in an integrated on-chip optofluidic environment

**DOI:** 10.1515/nanoph-2024-0139

**Published:** 2024-05-20

**Authors:** Fengji Gui, Ronny Foerster, Torsten Wieduwilt, Matthias Zeisberger, Jisoo Kim, Markus A. Schmidt

**Affiliations:** The Department of Fiber Photonics, Leibniz Institute of Photonic Technology, Albert-Einstein-Street 9, 07745 Jena, Germany; Abbe Center of Photonics and Faculty of Physics, Friedrich-Schiller-University Jena, Max-Wien-Platz 1, 07743 Jena, Germany; Otto Schott Institute of Materials Research, Friedrich Schiller University Jena, Fraunhoferstr. 6, 07743 Jena, Germany

**Keywords:** nanoparticle tracking analysis, optical fiber, optofluidic device, flat field

## Abstract

Here, we present the concept of flat-field capillary-assisted nanoparticle tracking analysis for the characterization of fast diffusing nano-objects. By combining diffusion confinement and spatially invariant illumination, i.e., flat-fields, within a fiber-interfaced on-chip environment, ultra-long trajectories of fast diffusing objects within large microchannels have been measured via diffraction-limited imaging. Our study discusses the design procedure, explains potential limitations, and experimentally confirms flat-field formation by tracking gold nanospheres. The presented concept enables generating flat-fields in a novel on-chip optofluidic platform for the characterization of individual nano-objects for fundamental light/matter investigations or applications in bioanalytics and nanoscale material science.

## 1 Introduction

Micro- and nano-scale particles, formed naturally or artificially, play an important role in many fields of research and application. The detection and sizing of such nano-objects is a fundamental task in scientific analysis, for example in environmental monitoring [1], biochemistry [2], [3] and life science [4], [5]. In many applications, the objects of interest have dimensions below the diffraction limit (typically a few tens of nanometers) and must be suspended in liquids, resulting in rapid Brownian motion that can be described by the diffusion equation [6]. One approach to accurately characterize nanoparticles (NPs) in such liquid environments relies on optical techniques such as dynamic light scattering (DLS), which is based on analyzing the scattered light from an ensemble of diffusing NPs. Although used successfully, the main drawback of DLS is that its sensitivity is dominated by large NPs, even though they represent only a small fraction of the size distribution.

Nanoparticle tracking analysis (NTA) is an optical technique for characterizing NPs on the individual species level. The size of the NP is calculated via statistically analyzing the trajectory of an individual object and applying the Stokes–Einstein relation [6]. NTA enables real-time monitoring and accurate diameter distribution measurement (10 nm–1 µm), with applications in life science [2], nanotechnology [7], and nanomedicines [8], [9]. Note that unlike DLS, NTA focuses on individual trajectories, thus supporting single particle tracking [10]. Due to the statistical nature of the data analysis, achieving high accuracy in NTA generally requires long trajectories, i.e., a large number of frames per trajectory. Thus, obtaining sufficiently long trajectories is critical in NTA research, which is particularly challenging for fast diffusing objects.

In this context, fiber-assisted NTA (FaNTA) is an emerging scheme used in NTA research [11]. The idea is to illuminate the NPs diffusing in a liquid channel running along an optical fiber through the modal field and to detect the laterally scattered light with a microscope. In addition to advantages such as light line illumination and fast readout, the key feature of FaNTA is the restriction of NP diffusion to the fluidic channel. This keeps the NPs in the field of view for much longer times compared to free diffusion and enables tracking them for extremely long times: it has been reported that nanobore optical fibers (NBF) enable the observation of fast diffusing nano-objects (*D*_dif_ ≈ 6 μm^2^/s) over 100,000 frames (40 s), providing exceptional statistical accuracy in the data analysis [12]. Different types of FaNTA implementation have been realized, including 3D tracking inside modified step-index fibers [13] or NP sizing inside anti-resonant element fibers [14], reaching NP sizes as small as 9 nm [15]. The scheme has been recently expanded towards nanoprinted on-chip hollow-core waveguides that are locally structured [16].

One issue that can limit the use of FaNTA is that the guided modal fields vary spatially along the transverse direction [11]. For example, NBFs have an evanescent field in the water-filled nanochannel, which could lead to fluctuations in the measured scattered intensity as the nanoparticle transversely diffuses in the fluidic channel, which could interrupt the tracking.

One approach is to generate modes with flat fields inside the liquid channel. We have recently demonstrated an implementation of this idea by adjusting the refractive index (RI) of the liquid channel in NBFs to match the effective mode index [17]. The resulting Poynting vector distributions show no spatial dependence along all three spatial directions, yielding a liquid light strand that significantly reduces intensity fluctuations in NTA experiments. Other more recent experiments indicate the application of flat-fields in the context of sensing using planar photonic waveguide technology [18].

For NBFs, however, the light strand has a transverse extension of a few hundred nanometers only, which can influence the diffusion of the NP and requires a modification in the data analysis. The use of larger cores is critical as the flat field condition becomes more susceptible to structural irregularities up to an extent that cannot be controlled by fiber fabrication. This motivates the realization of concepts that allow flat fields to be maintained even with larger channel diameters.

This work introduces the concept of capillary-based flat-field NTA for the characterization of fast diffusing nano-objects. By restricting NP diffusion within microchannels and providing spatially invariant illumination, i.e., a flat field, in a fiber-interfaced on-chip environment, very long trajectories of fast diffusing objects have been measured and statistically analyzed. The study discusses the design and possible limitations, and experimentally verifies the concept of flat-field illumination by tracking fast diffusing gold nanospheres.

## 2 Concept and demonstration

### 2.1 Operational principle

**Figure 1:**
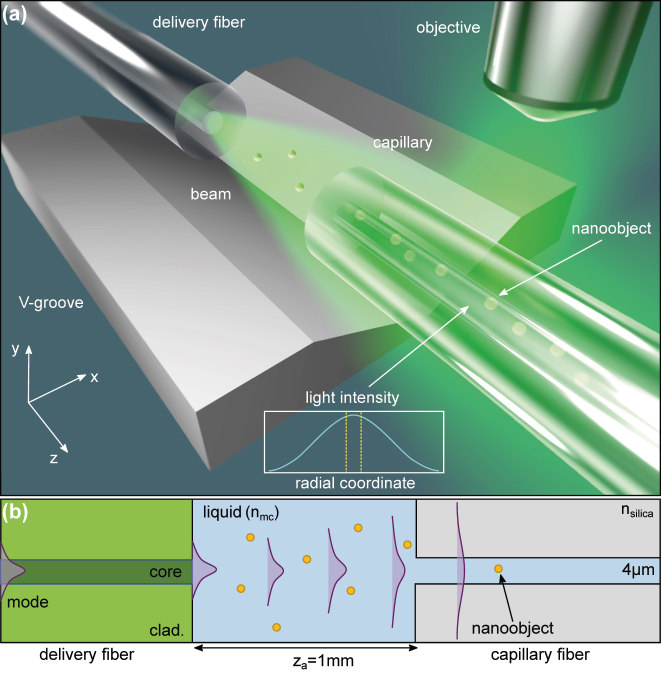
Flat-field capillary-assisted nanoparticle tracking analysis (CaNTA). (a) Illustration of the concept. The fluidic channel with gold nanoparticles is filled with a water-DMSO mixture that has a RI matching the cladding of the capillary. The inset shows the spatial distribution of the intensity of the Gaussian beam at the beginning of the capillary, showing an almost flat intensity distribution (the difference between the center and the edge is less than 1 %). (b) Sketch (top view) of the arrangement including the main elements and parameters.

The concept of flat-field capillary-assisted NTA (CaNTA) combines the idea of confining NP diffusion to a cylindrical channel with defined external illumination (Figure 1(a)): the idea is that the NPs diffuse inside a capillary and are illuminated by a defined Gaussian beam with a mode field diameter much larger than the diameter of the capillary hole. If the RI of the liquid inside the channel *n*_mc_ is matched to that of the material of the cladding *n*_clad_, no modes are formed inside the channel and the NPs experience an almost constant transverse intensity due to the very large extension of the illumination beam (Figure 1(b)).

### 2.2 Simulations

Key to the concept is the use of a defined, freely diffracting Gaussian beam inside a homogenous RI. In the arrangement discussed, this beam is generated by the mode at the end of an optical fiber. In case the RI contrast between core and cladding is sufficiently small, it can be shown that the behavior of the diffracting radiation can be approximated by a Gaussian beam (details in Supplementary Material Eq. (1)). To demonstrate the feasibility of the concept, the intensity distribution along the radial and longitudinal directions is simulated in the following, considering the experimental circumstances (details in Supplementary Material Figures S2 and S3).

**Figure 2:**
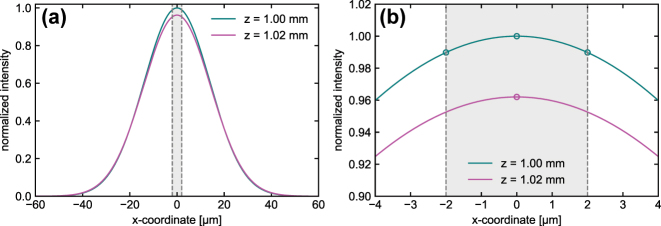
Properties of a diffracting Gaussian beam at a distance of 1 mm away from the beam waist. (a) Intensity distribution along a selected line along the radial direction for two distances from the fiber surface (cyan: *z*_a_ = 1 mm, magenta: *z*_b_ = 1.02 mm). The difference between the distances roughly refers to the diffusion length of a typical NP Δ*z* = *z*_b_ − *z*_a_ ≈ *L*_dif_ (details in main text). The gray area bounded by vertical dashed lines corresponds to the diameter of the microchannel used in the experiments (*D*_mc_ = 4 µm). (b) Close-up of the region close to the fluidic channel of the capillary. The cyan circles indicate the intensity in the center and at the side of the fluidic channel, while the magenta circle indicates the intensity in the center of the hole at *z*_b_. All simulation parameters are given in the main text.

The intensity distribution of a Gaussian beam (propagating inside a medium with a RI *n*_mc_ = 1.46 (close to the index of silica at visible wavelength), beam waist *ω*_0_ = 4.2 µm) is almost homogeneous along the radial direction (*xy*-plane) over the extent of the microchannel diameter considered (*D*_mc_ = 4 µm) if the distance between the delivery fiber and the NTA measurement area (*z*_a_ = 1 mm) is sufficiently large, as the intensity difference between center and edge of the channel is very small (<2 %, Figure 2).

The experimentally relevant intensity change along the longitudinal direction (*z*-axis) can be estimated from the diffusion length *L*_dif_ of a typical NP and the measurement time (*τ*_m_ = 65 s): for a single NP of diameter *d* = 50 nm diffusing in a liquid medium (viscosity *η* = 3 mPa·s), the Stokes–Einstein relation yields a diffusion coefficient of *D*_dif_ = 2.86 μm^2^/s, which corresponds to a diffusion length of $L_{\text{dif}}=\sqrt{2D_{\text{dif}}\tau_{\text{m}}}\approx 19$ µm. The longitudinal intensity decrease over such a length at the distance *z*_a_ is small Δ*I*(*L*_dif_) ≈ 4 % and can practically be neglected in NTA experiments. It should be noted that *I* can be further reduced by increasing the distance between delivery and capillary fiber.

The working principle of the concept relies on using a liquid medium whose RI *n*_*l*_ must match that of the cladding *n*_clad_ at the operation wavelength to avoid mode formation, which would lead to an uneven field inside the microchannel. This leads to the flat-field condition Δ*n* = *n*_mc_ − *n*_clad_ = 0 with the RI-mismatch Δ*n*. To reveal the impact of a possible RI mismatch on the propagation of light inside the microfluidic channel and the cladding, finite-element simulations (COMSOL Multiphysics 5.2) were performed on a configuration that is comparable to the experiments performed (cladding material: silica with RI *n*_silica_(*λ*_0_ = 0.532 µm) = 1.4607). In the simulation, a plane wave with a *k*-vector along the *z*-direction was defined as the excitation field at the input of the simulation area, and the scattered fields from the liquid-filled capillary with different Δ*n* were studied (more details in the Supplementary Material).

**Figure 3:**
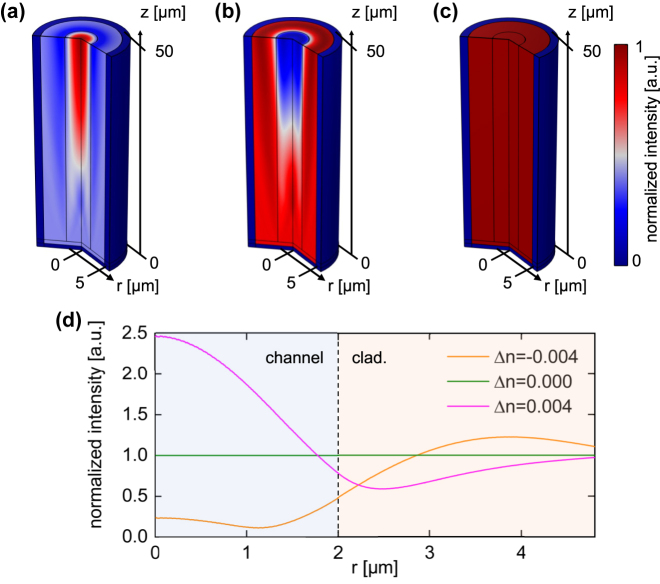
FEM-simulation of the intensity distribution inside a selected section of the capillary (*z*_*a*_ < *z* < *z*_*a*_ + Δ*z*) for three RI scenarios (characterized by *n*). (a) Δ*n* = 0.004, (b) Δ*n* = −0.004, (c) Δ*n* = 0. (d) Corresponding radial intensity distributions at *z* = *z*_*a*_ + Δ*z*. All distributions shown in the figure are normalized to the value of the RI-matched case.

In the following, the intensity distribution in the spatial domain Δ*z* = 50 μm at a distance *z*_a_ is investigated for relatively small RI mismatches, which may be relevant due to practical limitations. Two cases can be distinguished:

1. For Δ*n* > 0 total internal reflection is present and gives rise to guided modes in the microchannel (Figure 3(a)), which show a pronounced intensity variation both axially and transversely.
2. The complementary case (Δ*n* < 0) leads to the formation of so-called leaky modes, which dissipate power transversely during light propagation (Figure 3(b)). In the ray picture, this behavior can be explained by a zigzagging beam inside the microchannel with less-than-unity reflection at the liquid/silica interface.

For both scenarios, intensity variations occur even at relatively small RI mismatches (Δ*n* = ±0.004). In contrast, there is no visible intensity change in the case of matched RIs (Figure 3(c)). To further illustrate the impact of the RI mismatch, Figure 3(d) shows the radial intensity distributions at the end of the simulation volume at *z* = *z*_a_ + Δ*z* along a selected line. For Δ*n* = 0.004, the intensity in the center of the capillary is about 2.5 times greater than at the liquid/wall interface. In the complementary case (Δ*n* = −0.004), the intensity in the center of the system is significantly lower than at the corresponding interface. This discussion clearly reveals that the RI of the liquid used in the experiments must be controlled precisely to minimize intensity variations.

### 2.3 Experiments

Gold nanospheres (average diameter 50 nm, concentration 8·10^8^ NPs/ml, [nanoComposix]) were used as NPs to confirm flat-field formation in the experiments. Based on the results of a previous study [13], a mixture of water and Dimethyl sulfoxide (DMSO) was used as the host liquid, allowing the RI to be precisely adjusted by changing the mixing ratio (details in the Methods section). In the current experiments, a mixing ratio (in weights) of 16.9 % water and 83.1 % DMSO mixture was chosen, giving an RI of *n*_mc_ ≈ 1.4607, which corresponds to the value for silica [19]. Note that the use of DMSO for biological specimen requires extra caution due to possible cytotoxicity [20].

The experimental setup consists of a low-NA delivery fiber (NA = 0.05) and a capillary (*D*_mc_ = 4 µm), both aligned in an etched V-groove on a silicon chip and exposed to the liquid environment mentioned above (more details in Methods section). The distance between the fiber and the capillary was set to *z*_a_ = 1 mm, considering the properties of the delivery fiber. The arrangement is exposed to the NP solution, which is filled into the central hole of the capillary (hole diameter: *D*_mc_ = 4 µm, outer diameter 125 µm) by the capillary effect [21]. Typical filling times are of the order of a few minutes (more details can be found in the Supplementary Information of Ref. [13]). Note that the experimental assembly was subjected to a plasma treatment before loading the sample solution to improve surface wettability and eliminate the risk of bubble formation. The diffusion of the NPs along the longitudinal direction (*z*-direction), confined by the microchannel, is measured using a microscope that detects the laterally scattered light using a fast camera (frame rate *ν* = 1 kHz). A tracking area close to the entrance of the capillary was selected to avoid the disturbing by the reflected beams from the outer surface of the capillary fiber.

**Figure 4:**
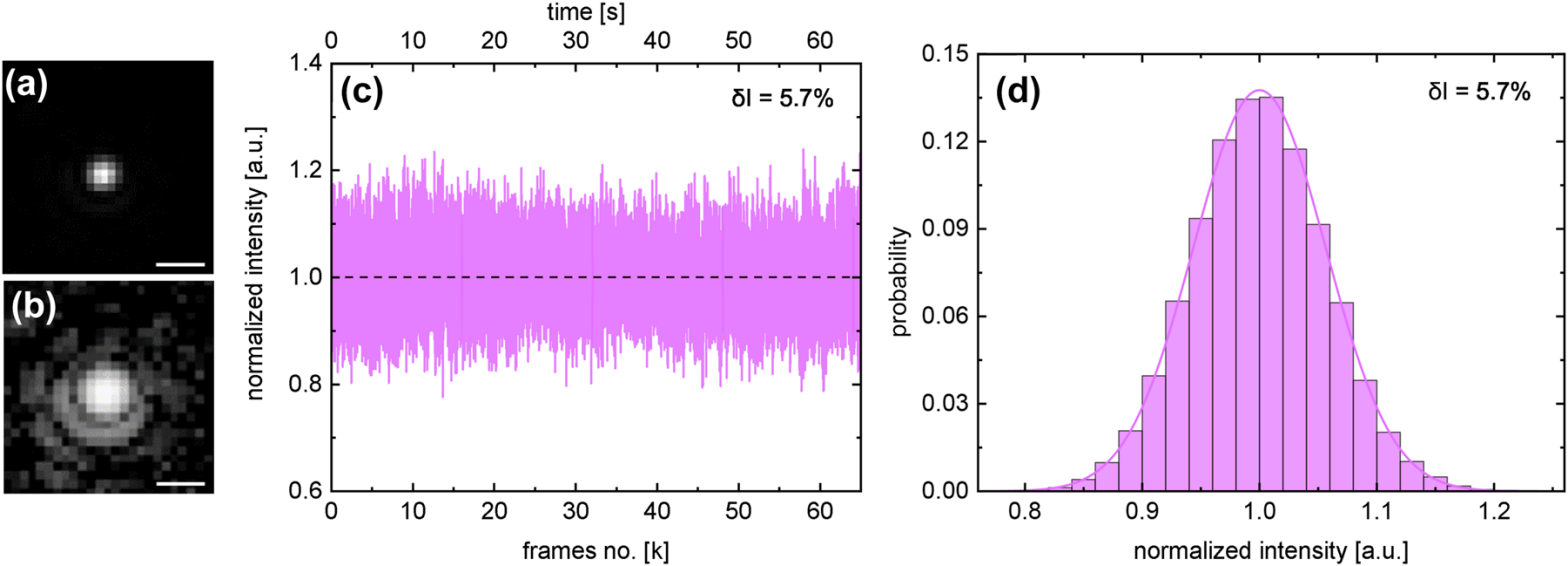
Statistical analysis of time-varying scattering intensity of a nanoparticle diffusing inside the illuminated capillary. (a and b) Close-up views of a NP in one selected frame using the mentioned imaging system ((a) linear scale, (b) logarithmic scale). The Airy rings are clearly visible, revealing that the imaging is diffraction-limited. The scale bar is 2 µm. (c) Measured temporal variation of the scattering intensity of the NP (*d* = 50 nm) diffusing inside the microchannel. (d) Histogram of *I*_*s*_ with a Gaussian fitting. Note that the intensities in (c) and (d) have been normalized to the value of the average intensity.

Two selected images of frames before and after the liquid exposure are shown in Supplementary Material Figure S5. The quality of the imaging system is illustrated by the image shown in Figure 4(a and b). Here, the Airy pattern is clearly visible, indicating that the imaging system is diffraction limited (c.f. [14]). Note that the influence of the plasmonic properties of the NP used on the optical detection is minimal, since the variation of the scattering cross-section *σ*_scat_ within the spectral range of interest (the visible spectral domain, 500 nm $<\lambda_{0}<$ 700 nm) is less than one order of magnitude. The variation of the scattered intensity is therefore small when the operating wavelength is changed. The potential influences of photon pressure and induced pressure have been quantified by calculating critical intensities and comparing them to the system described in this study (Section 11 of the Supplementary Material). This analysis indicates that these factors are insignificant in the context of the experiments described herein.

### 2.4 Data analysis

**Figure 5:**
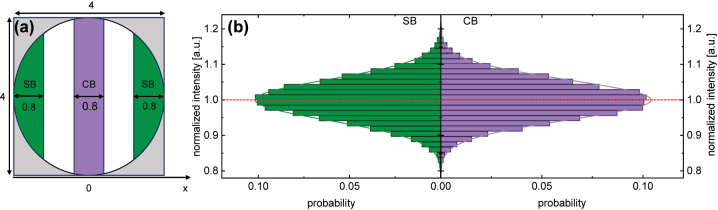
Verification of the flat-field concept by statistical analysis of the time-varying scattering intensity in selected spatial domains of the microchannel. (a) Cross-section of the capillary with the areas in which the scattering intensity is binned and statistically analyzed (purple: central bin [CB], dark green: side bins [SBs]). The numbers are given in µm. (b) Histograms of the intensity distributions in the CB and the SBs. The colored lines indicate the corresponding continuous curves from a Gaussian fitting. The number of data points used for the histograms is *N*_CB_ = 17,632 and *N*_SB_ = 14,821 for the CB and SBs, respectively.

To get information on the field profile inside the microchannel, the scattering intensity of the diffusing NPs has been evaluated in selected regions of the microchannel (details can be found in the Method section and Ref. [17]). Specifically, the domain of the fluidic channel was transversely divided into a central bin (CB) and two intensity side bins (SBs, Figure 5(a)). Then the measured intensity values are binned within these spatial domains and the corresponding occurrence probabilities are plotted in histograms. To extract the key parameters of the individual distribution, Gaussian distributions have been fitted to the histograms. The diameter of the NPs of interest was determined using mean-square-displacement (MSD) analysis, i.e. a statistical analysis of the trajectory of the NPs resulting from Brownian motion (Figure 4). Details of MSD analysis, which is well-established in the context of FaNTA, can be found in several of our previous publications [12].

## 3 Results

### 3.1 Experimental demonstration: analysis of the entire trajectory

To verify the flat-field concept, the trajectory of a single NP was recorded and the temporal variation of *I* was statistically analyzed (Figure 4(c)). As mentioned above, high statistical significance requires the recording of trajectories with a very large number of frames *N*, which can be achieved by restricting the diffusion to a defined spatial region. In the context of the FaNTA, this condition is enforced by the microchannel environment [11], [12]. In the present experiment, the characteristic feature led to a long measurement time (*τ*_*m*_ = 65 s) at a high frame rate (*ν* = 1 kHz), resulting in a trajectory consisting of a very large number of frames (*N* = 65,000).

Analyzing the distribution of the time-varying scattering intensities results in an overall symmetrical Gaussian distribution (Figure 4(d)), which indicates a constant intensity distribution and thus flat fields within the microchannel. This can also be seen in the visual inspection of the analyzed movies, which show hardly any temporal variation of the scattered intensity (an example of a 1-min trajectory is uploaded as Visualization 1). It should be noted that due to intrinsic fluctuations, a Gaussian distribution can theoretically be expected even for a constant illumination [17]. Another indication of flat fields is the symmetry of the distribution in relation to the mean value of the scattering intensity $\bar{I}$ . In principle, a deviation of the intensity distribution in the microchannel from a flat field (e.g., evanescent decay) would lead to an asymmetric distribution, which was not observed in the present experiments. A further indication of the presence of flat fields results from the low value of the relative standard deviation of $\delta I=\sigma_{I}/\bar{I}=5.7$ % (*σ*_*I*_: measured standard deviation). It can be assumed that this value essentially results from statistical fluctuations of the measurement approach and not from a variation in the intensity distribution in the microchannel.

### 3.2 Experimental demonstration: analysis in the two selected spatial domains

The next step in the analysis relies on comparing the occurrence probabilities of the scattered intensities in the two spatially separated regions named CB and SBs (*I*_*i*_ with *i* = CB, SB, Figure 5(a and b)). All intensities shown in the following are normalized to the maximum of the intensity of the Gaussian fit in the CB, i.e., $I_{i}\to I_{i}/I_{\text{CB}}^{\text{max,G}}$ . Several important characteristics can be extracted from this comparison that support the observation of flat-field formation (all numbers discussed here are summaries in Table 1): first, the probability distributions are symmetric regarding the corresponding average probability, which according to the discussion of the previous section indicates a constant intensity within the respective bin. This point is supported by the fact that the FWHM extents of the distributions are comparable $\left(\Delta I_{\text{CB}}^{\text{FWHM}}\approx \Delta I_{\text{SB}}^{\text{FWHM}}\right)$ . An even stronger argument for flat-fields is that the peak intensities of the Gaussian fits of both distributions have approximately the same peak intensity $\left(I_{\text{SB}}^{\max}\approx I_{\text{CB}}^{\max}\right)$ , as indicated by the red dashed line in Figure 5(b)). This point can be qualified by inspecting the difference between the peak intensities of the CB and SB $\left(I_{i}^{\max}\right)$ defined as $\delta I_{\max}=\left(I_{\text{CB}}^{\max}-I_{\text{SB}}^{\max}\right)/I_{\text{CB}}^{\max}$ , which is *δI*_max_ = −0.23 % for the experiments reported here.

**Table 1:** Key parameter of data analysis that is related to the statistical analysis of scattered intensity in selected regions of the microchannel cross-section.

Parameters	Symbols	Unit	Value
Peak intensity of CB	$I_{\text{CB}}^{\max}$	1	1.00001
Peak intensity of SB	$I_{\text{SB}}^{\max}$	1	1.00235
FWHM of CB	$\Delta I_{\text{CB}}^{\text{FWHM}}$	1	0.13351
FWHM of SB	$\Delta I_{\text{SB}}^{\text{FWHM}}$	1	0.13529
Peak intensity difference	*δI*_max_	%	−0.23
Relative standard deviation of intensity	*δI*	%	5.7

### 3.3 Mean-square-displacement analysis

**Figure 6:**
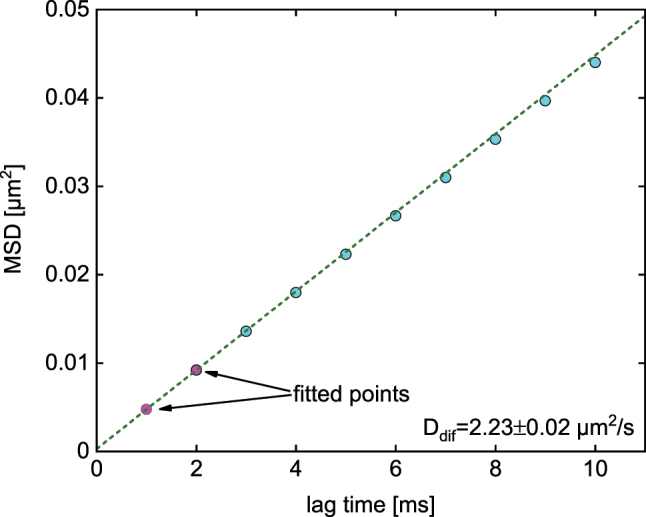
Application of the flat-field concept to determine the hydrodynamic diameter of the investigated NP using MSD analysis. MSD as a function of lag time (points: measured values). The dashed dark green line refers to a linear fit of the first two data points (magenta dots).

The advantage of flat-field illumination and the resulting long trajectory length was used here to determine the hydrodynamic diameter *d*_h_ of the investigated NP by MSD analysis of the Brownian motion (Figure 6). A detailed description of this data processing method in relation to FaNTA can be found here [12]. The diffusion coefficient of the nanosphere was determined by fitting the MSD values of the first two lag times, (*D*_dif_ = 2.23 μm^2^/s) and the hydrodynamic diameter was calculated using the Stokes-Einstein relation *d*_h_ = *k*_b_ *T*/(3*πη*(*T*)*D*_dif_). Note that in MSD analysis, a single lag time does not correspond to a single time, but refers to a statistical average of the position differences over the entire trajectory. For example, the shortest lag time Δ*t* results from averaging over *N* − 1 values and thus leads to a high statistical significance. The significance decreases with longer lag times due to the smaller number of values considered. As detailed in one of our previous works [12], when the localization error in tracking experiments is negligible – as in the present study – the first two lag times generally achieve the greatest statistical significance and provide the most accurate determination of the diffusion coefficient through linear fitting the two experimental data points (c.f. [22]). Note that including more lag time typically reduces the accuracy of statistics, especially in situations where diffusion is spatially restricted. Considering the ambient temperature (*T* = 293.15 K), the viscosity of the DMSO/water mixture is approximately *η* = 3.3 × 10^−3^ Pa·s [23], yielding a hydrodynamic diameter of *d*_h_ = 58.4 ± 0.6 nm. The measured *d*_h_ is consistent with the expected value of the used NPs which have a mean hydrodynamic diameter of 59 nm.

It should be noted that the influence of the confinement of the microchannel on diffusion can be neglected in this work, since the first four lag times show a clear linear dependence. Furthermore, the hindrance factor, which describes the influence of the confinement on the diffusion coefficient, was determined for the present geometry, yielding *R*_ave_ = 1.066 (a detailed analysis of confined diffusion in the context of FaNTA can be found here [24]). As this factor is in the range of 1, the value of *d*_h_ remains almost unchanged, i.e., free diffusion can be assumed (further details on this argumentation can be found in the Supplementary Information of Ref. [15] in Sections 15 and 16). Note that the work of Nitsche and Balgi [25] shows that the confinement of NP diffusion can be neglected when the ratio of NP to microchannel diameter is very small. Since the experiments show no average drift of the NPs along the fiber, an influence of the thermophoretic effect can be excluded.

## 4 Discussion

The concept presented combines the confinement of NP diffusion to a microchannel with flat-field illumination, resulting in very long measurement times and thus excellent statistics, outperforming many other NTA implementations. Crucially, the NP is always visible and never experiences low light intensity. Here, the Cramers–Rao bound [14] shows that such long trajectories lead to significantly better statistics, as the standard deviation of the diffusion coefficient depends on the number of frames $\left(\sigma_{\text{D}}\propto 1/\sqrt{N}\right)$ . In addition, the use of long tracks makes it possible to measure dynamic processes of single species that occur at any time. It should be noted that the described concept can also be employed in the case of transparent NPs, as it does not depend on plasmonic effects. However, to compensate for the smaller scattering cross-section, a larger NP diameter is typically required. Another advantage of the flat-field illumination lies within the analysis of polydisperse specimens, which is challenging for NTA due to the strong dependence of the scattering intensity on NP diameter: the smallest NP *d*_min_ should be visible above the noise floor of the camera, while the largest NP *d*_max_ should not impose saturation. For example, assuming a camera pixel noise floor of *I*_min_ = 200 and a saturation limit of *I*_max_ = 65,000 (16 bit), the ratio of detectable NP diameters is *δd* = (65,000/200)^1/6^ ≈ 2.6 which is high compared to other waveguides that typically have center/edge intensity ratios of 200:1, making the investigation of polydisperse specimen substantially challenging. The experimental setup is chip-integrated and fiber-coupled, thus including a high level of microfluidic integration. The minimum and maximum NP concentrations that can be measured with the experimental setup discussed in this study are *c*_min_ = 3.8·10^8^ NPs/ml and *c*_max_ = 25·10^8^ NPs/ml for a total measurement time of *τ*_m_ = 65 s (details can be found in the Supplementary Material, Section 10).

One aspect that may affect the performance of the concept is the temporal variation of the intensity before the capillary input, which is associated with the scattering of light of diffusing NPs that are located between delivery fiber and capillary. The impact has been investigated by analyzing the scattered intensity of a static NP fixed inside the microchannel. As shown in the Supplementary Material Figure S6, the time-dependent intensity in the microchannel is stable (variations *δI* ≈ 1 %) and the light scattering in the space between fiber and capillary can be safely ignored.

Comparable flat-field experiments have been performed by the authors in another study using a novel type of optical mode [17]. Here, the capillary-based concept presented offers the advantage of a larger hole diameter, simplifying data analysis, as the influence of the channel on the diffusion coefficient can be neglected (for the given frame rate). In addition, the filling of the microchannel is much faster, as suggested by Washburn’s equation [21]. An example calculation is shown in Figure S8 in the Supplementary Material. In principle, very short capillary sections can be used for CaNTA because, unlike NBF, no transition region is required in which the mode forms. A capillary length of 1 mm is sufficient for the experiments presented here, whereas longer sections are required for waveguides. Simulations of the transverse intensity distribution, including the thermo-optic response of the liquid mixture (see Supplementary Material, Section 5) show that (i) temperature variations of the order of 1 °C lead to acceptable variations in the field distribution and (ii) lower (higher) temperatures impose a guided (leaky) mode type behavior due to the increased (decreased) refractive index.

An essential requirement for the approach described here is that the RI of the liquid must match the RI of the capillary, limiting the choice of possible liquids (for instance, the resulting intensity distribution in the case of water is shown in Figure S2 in the Supplementary Material). This is a clear incentive for further research to expand the portfolio of possible liquids. In addition to other glass materials, the implementation of the concept using planar technology seems to be important here, as materials inaccessible to fiber optics, such fluorine materials (e.g., MgF_2_ [26]), or porous layers (e.g., mesoporous silica [27]) can be used. An alternative approach includes the utilization of microstructured capillaries with holey or structured cladding. In this context, it is crucial to elucidate the potential impact of additional interfaces on optical imaging and, consequently, the tracking process.

The concept presented is not limited to optical fibers and can, in principle, be transferred to other geometries. For example, the delivery fiber can be replaced by a planar waveguide and the capillary by an optofluidic channel, further increasing the level of integration in terms of on-chip integration. In addition, by functionalizing the end surface of the delivery fiber, substantially more complex beam profiles can potentially be created, allowing flat field formation for even larger microchannel diameters. It has been shown that 3D nanoprinting can be used to create complex phase holograms [28], [29] or metastructures [30], [31] on fibers. Possible relevant profiles are super-Gaussian beams [32] or flat-top patterns [33] that can be created using nanostructures. Overall, the presented approach represents an unexplored contribution to the field of lab-on-chip and lab-on-fiber technology [34], [35].

## 5 Conclusions

Here we introduce the concept of flat-field capillary-assisted NTA (CaNTA) for the characterization of fast diffusing nano-objects. By illuminating a NP solution-filled microchannel with a RI matched to the capillary glass by a large Gaussian beam, fields with no spatial dependence, i.e., 3D flat fields, are obtained within the fluid. The unique combination of diffusion confinement and flat-field illumination allows the recording of ultra-long trajectories (65 s) of fast diffusing NPs (diameter 50 nm) at very high frame rates (1 kHz) within a comparatively large microchannel (4 µm) while providing diffraction limited imaging. Capillary and Gaussian beam delivery fiber are interfaced on a silicon chip, thus achieving a high level of on-chip optofluidic integration. Our study discusses the design procedure, explains potential limitations through simulations, and experimentally confirms flat field formation within the microchannel by tracking gold nanospheres, including sophisticated data analysis.

The presented concept enables the generation of flat fields in an on-chip environment and thus offers a novel integrated optofluidic platform for the analysis of individual NPs. Applications can be found in various fields such as bioanalytics (e.g., characterization of nano-objects), nanoscale material science (e.g., analysis of chemical reactions or nanorheological aspects), while fundamental studies of light-matter interactions (e.g., nanoscale refractive index measurements) can be performed at the single species level.

## 6 Methods and materials

### 6.1 Nanoparticle solution preparation

Gold nanopsheres (ultrauniform 50 nm, nanoComposix) dispersed in a mixture of water and DSMO were used as NPs in this study. By varying the mixing ratio, the RI of the binary liquid was precisely adjusted here to achieve the flat-field condition *n*_mc_ = *n*_clad_ = *n*_silica_. The applicability of this liquid for NTA experiments was confirmed in one of our previous studies [13], showing that DMSO-water mixtures have no detrimental impact on diffusing gold NPs and NTA experiments, as long as the correct viscosity (*η* = 3.3 × 10^−3^ Pa·s @ *T* = 293.15 K) is considered in the Stokes-Einstein equation.

### 6.2 Experiments

The experimental setup is shown in Figure S1 in the Supplementary Material. Delivery (in-house made single-mode fiber with the cut-off wavelength of 457 nm, NA = 0.05) and capillary fibers (*D*_mc_ = 4 µm, made from silica [F300, Heraeus]) were aligned inside a V-groove etched into a silicon chip (both have an outer diameter of 125 µm), with the delivery fiber fixed by using UV curing epoxy (Vitralit^®^1605). The distance between the fiber and the capillary surface was set to *z*_a_ = 1 mm, which can be adjusted by a 3D-stage during the experiment, according to the estimation discussed above. To prevent bubble formation, a plasma treatment of 100 W for 1 min was performed prior to liquid exposure. The area between the fibers was immersed in the prepared NP solution and covered with a glass slide to improve the image quality. For the tracking experiments, green laser light (Coherent Verdi G, *λ* = 532 nm) was coupled into the delivery fiber, yielding a power of *P*_out_ = 17 mW at the output of the delivery fiber. The NPs were imaged using a 10× objective (Olympus Plan Achromat, numerical aperture 0.25, depth of field 10 µm) and recorded by a CMOS camera (Basler acA4096-40 um) with a field-of-view of the captured frames of (30 × 600) pixel. In the tracking experiment, the frame rate and exposure time were *ν* = 1 kHz and *τ*_e_ = 0.2 ms, respectively. In this study, the trajectory of a single NP was recorded (number of frames: *N* = 65,000, total measurement time: *τ*_m_ = 65 s).

### 6.3 Data analysis

The trajectories of the NPs were extracted from the recorded images using the Python-based package *Trackpy*. The fluidic channel was divided into the center and side bins (CB and SBs) according to the x-positions (Figure 5(a)). To analyze the mode distribution in the channel, the scattered intensities in the CB and SBs were plotted as histograms and fitted by Gaussian distributions. The diameter of the NP of interest was calculated using mean-square-displacement (MSD) analysis by statistically analyzing the trajectory of the NPs, i.e., the Brownian motion (Figure 6). Details of MSD analysis in the context of FaNTA can be found in several of our previous works [12], [13].

## Supplementary Material

Supplementary Material Details
