# Supplementary material for: Capillary-assisted flat-field formation: a platform for advancing nanoparticle tracking analysis in an integrated on-chip optofluidic environment
Source: Nanophotonics. 2024 May 20;13(17):3135–45. doi: 10.1515/nanoph-2024-0139 (PMC11501659; doi:10.1515/nanoph-2024-0139)
Supplement: Supplementary file 1 — Supplementary Material Details [file j_nanoph-2024-0139_suppl_001.pdf]

Supplementary Material: Capillary-Assisted Flat-Field Formation: a Platform for Advancing Nanoparticle Tracking Analysis in an Integrated On-Chip Optofluidic Environment

Fengji Gui[1], Ronny Foerster[1], Torsten Wieduwilt[1], Matthias Zeisberger[1], Jisoo Kim[1], and Markus A. Schmidt[1,2,3,*]

[1]The Department of Fiber Photonics, Leibniz Institute of Photonic Technology, Albert-Einstein-Street 9, 07745 Jena, Germany
[2]Abbe Center of Photonics and Faculty of Physics, Friedrich-Schiller-University Jena, Max-Wien-Platz 1, 07743 Jena, Germany
[3]Otto Schott Institute of Materials Research, Friedrich Schiller University Jena, Fraunhoferstr. 6, 07743 Jena, Germany;
[*]E-mail: markus-alexander.schmidt@uni-jena.de

1 Gaussian beam

The optical beam that illuminates the capillary and the microchannel can be approximated as a Gaussian beam (Fig. 1(b) in main text). The intensity distribution of the beam can be expressed as follows:

$$I(r,z) = I_0 \left[\frac{\omega_0}{\omega(z)}\right]^2 \exp\left[-2r^2/\omega^2(z)\right] \tag{1}$$

where $\omega(z) = \omega_0\sqrt{1+(z/z_\mathrm{R})^2}$ is the beam radius as the function of axial position, and $z_\mathrm{R} = \pi\omega_0^2 n/\lambda$ is the Rayleigh range. ω_0 is the radius of the beam waist, which is 4.2 µm (the modal radius of the launching fiber), $n_\mathrm{mc} = 1.4607$ is the refractive index of the liquid and $\lambda = 532$ nm is the operation wavelength.

2 Experimental setup

The experimental setup for characterizing the fiber-assisted optofluidic device is shown in Fig. S1. Light from a cw laser (Coherent Verdi G, 532 nm) is delivered via a single-mode fiber (Thorlabs, S405-XP, black), which was spliced to an in-house made low-NA fiber containing the polarization controller (green). The light (polarized in the x-direction) was then coupled fiber-to-fiber into an identical delivery fiber (low-NA fiber, green) that carries the power to the chip region. Taking into account the coupling losses at the various junctions, the power at the output of the delivery fiber is estimated to be about $P_\mathrm{output} = 17$ mW. The sample of NP suspension is placed directly on the chip and is filled into the microchannel via capillary force. The end of the capillary is blocked by glycerin after the filling is completed to avoid additional liquid flow. A glass coverslip is used to enhance the imaging quality. The NTA experiments are performed by using a standard optical microscope with the 10X objective lens ($NA = 0.25$, Olympus Plan Achromat). Nanoparticles inside the fluidic channel were imaged through a CMOS camera (Basler

acA4096-40um) with the height and width of the recorded frames being (30 × 600) pixel. The frame rate and exposure time were 1000 Hz and 0.2 ms, respectively.

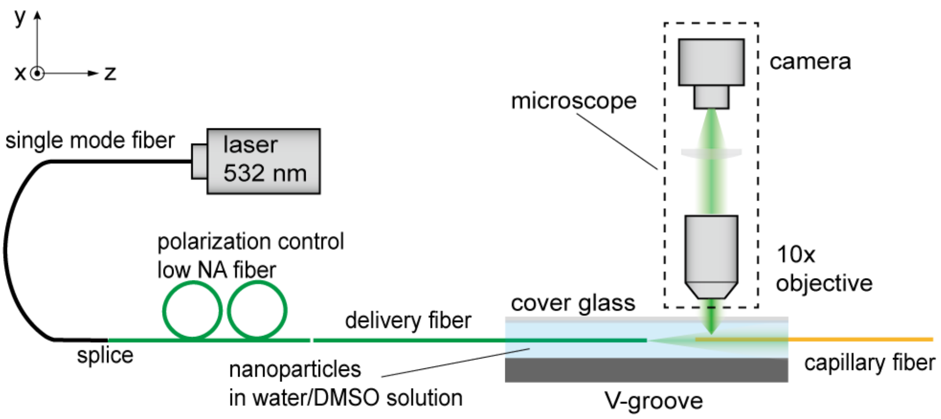

Figure S1: Experimental setup for fiber-assisted nanoparticle tracking analysis using a divergent Gaussian beam illumination. The length of the delivery fiber was 3 cm in the measurement. The polarization is parallel to the x-direction. Single mode fiber: S405-XP (Thorlab); delivery fiber: NA=0.05, mode diameter 8.4 µm.

3 Description of simulation of light propagation in the RI-mismatched situation

The modal fields in the capillary are simulated using a 2D axisymmetric model in Comsol Multiphysics. Without loss of generality, plane waves are defined as the excitation fields in the simulation domain, and the scattered fields within the fluidic microchannel are computed. In the simulation, the fluid domain extends over a distance of 1 µm outside the capillary as the starting region, so the excitation fields in the simulation can be written as piecewise expressions using the Fresnel equations [1]: (i) fluid part: $E_1 = E_0\exp(-ik_1z) + r_0E_0\exp(ik_1z)$; (ii) capillary part: $E_2 = t_0E_0\exp(ik_2z)$, where $r_0 = (n-n_\mathrm{c})/(n+n_\mathrm{c})$ is the reflection coefficient and $t_0 = 2n/(n+n_\mathrm{c})$ is the transmission coefficient at the liquid-silica interface. n and n_c are the refractive indexes of the liquid and capillary, respectively. E_0 is the amplitude of the plane wave, $k_1 = 2\pi n/\lambda$ and $k_2 = 2\pi n_c/\lambda$ are the wave numbers in the liquid and in the microchannel fiber, respectively. Perfectly matched layers (PML) are used as the outer boundaries to simulate the infinite domains. The smallest mesh size is set as $\lambda/15$, which leads to 1 hour of the simulation time.

4 Light propagation in the capillary

A 3D view of the intensity distribution of the mode for the case of water ($n_\mathrm{mc} = 1.3337$ [2]) is shown in Fig. S2(a), with the corresponding distribution along a selected line in the radial direction shown in (b) (measured at the position of $\Delta z = 50$ µm). The appearance of a mode is clearly visible due to reflection at the liquid/glass interface. Note that the intensity shows both azimuthal and longitudinal dependence, as indicated by the fact that the light intensity at the edge of the microchannel has decreased by 90% compared to the centre. This will limit the measurement capabilities of NTA, as the scattered intensity of the diffusing nanoparticles will fluctuate strongly with the Brownian motion. Furthermore, the cases of relatively small differences in the refractive index between the liquid and the capillary Δn (ranging from -0.008 to 0.008) are shown in Fig. S3. The simulations show that, depending on whether $\Delta n < 0$ or $\Delta n > 0$, leaky or guided modes are formed in the microchannel, both of which should be avoided if flat-field illumination is required.

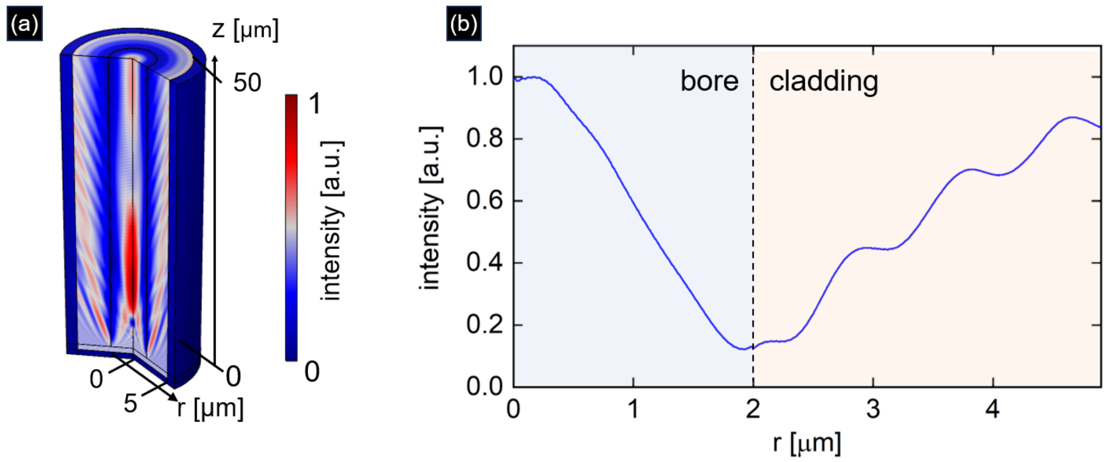

Figure S2: Simulations of the intensity distribution in the microchannel of the capillary in case water is considered as liquid medium. (a) 3D view of the intensity distribution in the capillary. (b) Distribution along a selected line in radial direction at $\Delta z = 50$ µm.

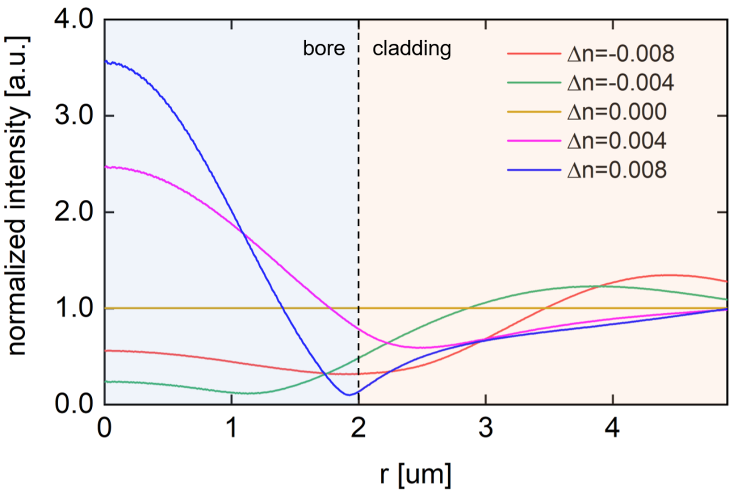

Figure S3: Simulation of the radial intensity distribution in the capillary with the refractive index of the liquid, mismatching that of the silica capillary ($n_{\text{silica}} = 1.4607$).

5 Thermo-optic response

To uncover the impact of temperature on the electromagnetic field distribution, additional Finite-Element simulations of the spatial intensity distribution have been performed, similar to those shown in Fig. 3 of the main text, now including the temperature variation of the refractive index of the liquid mixture. In accordance with the considerations of Ref. [3] the following equation for the material dispersion of the liquid mixture was used to calculate the temperature-related behavior (c.f. Supplementary Information of Ref. [3]):

$$n(\lambda_0, c_{\text{m}}) = n_0(\lambda_0, c_{\text{m}}) + \Delta n_T (T - T_0) \tag{2}$$

with the operation wavelength $\lambda_0 = 532$ nm, the concentration (weight) ratio of water and DMSO $c_{\text{m}} = 83.1$ wt% (e.g., $c_{\text{m}} = 0$ wt%: 100% water, $c_{\text{m}} = 100$ wt%: 100% DMSO) and the measured material dispersion of the mixture $n_0(\lambda_0, c_{\text{m}}) = 1.4587$ at $T = T_0 = 25$°C. The experiment in the present work was carried at 20°C. The temperature-related correction term is given by [4]

$$\Delta n_T = -10^{-4} \cdot \frac{dn}{dT} = -10^{-4} \cdot (-0.000116 \cdot c_{\text{m}}^2 + 0.0429 \cdot c_{\text{m}} + 1.0837) \tag{3}$$

The results of the numerical simulation (Fig. S4) clearly show that (i) temperature variations in the order of $\pm1°C$ lead to acceptable variations in the intensity distribution, and (ii) lower (higher) temperatures impose a guided (leaky) mode type behavior due to the increased (decreased) refractive index of the liquid mixture.

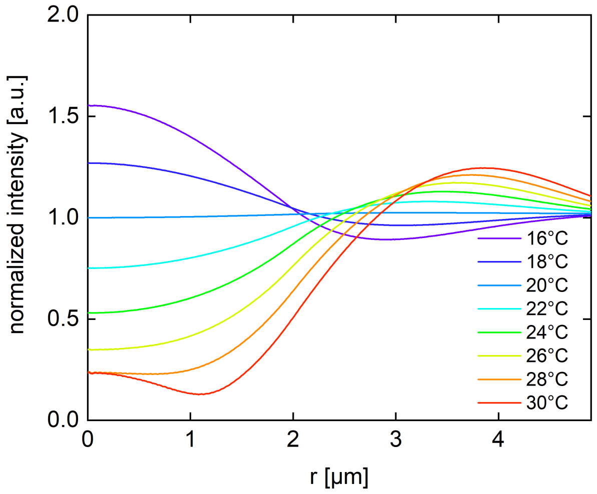

Figure S4: Finite-Element simulations of the transverse intensity distribution at $z = z_a + \Delta z = 1050$ μm for different temperatures (simulation procedure is explained in the main text). All distributions shown in the figure are normalized to the value of the RI-matched case.

6 Microscopic image of the working device

A microscopic image of the fiber-assisted optofluidic device (before loading the sample) is shown in Fig. S5(a). The capillary fiber is movable along the z-direction on the V-groove, and the distance to the delivery fiber is $z_a = 1$ mm. A representative image of

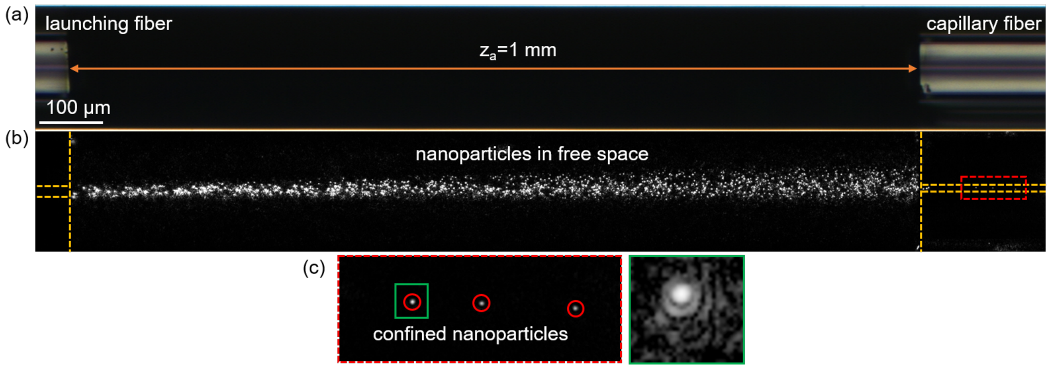

Figure S5: Representative images of the fiber-assisted optofluidic device and images taken during performing the experiments. (a) Microscopic image of the device before filling the liquid. The distance between the delivery fiber and the capillary is around 1 mm. (b) Image after filling in the liquid sample and switching on the illumination light. (c) Enlarged view of nanoparticles diffusing in the fluidic microchannel of the capillary. The intenisty of the nanoparticle framed by the green box is displayed in logarithmic scale on the right-handed side.

the device and the imaged nanoparticles suspended in the RI-adjusted liquid are shown in Figs. S5(b) and (c), respectively. It is worth noting that the light field becomes more

flat when the propagation distance of the beam is larger, while the illuminating intensity will reduce accordingly, e.g., the central intensity drops to 1.2% after a propagation of 1 mm in this case. The tracking area of nanoparticles in this work was selected close to the input part of the capillary, which is shown as the red dashed box in Fig. S5(b).

7 Intensity measurement of a static nanoparticle

To quantify the inherent noise of the optofluidic and imaging systems in this work, the scattered intensity of an immobile nanoparticle in the microchannel was measured. The intensity as a function of the frame index has a Gaussian distribution with a relative standard deviation of 1.1% (Fig. S6). This intensity fluctuation result from the scattering of the freely diffusing NPs diffusing between the delivery fiber and capillary (Fig. S5(b)), as well as the laser system and readout noise of the camera.

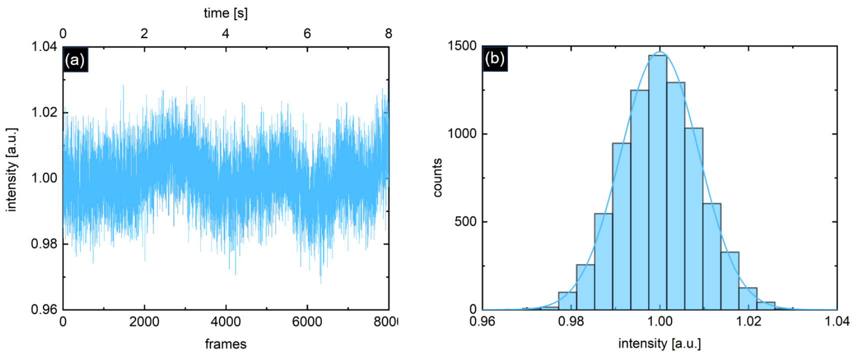

Figure S6: Temporal behavior of the intensity of a static nanoparticle in the fluidic microchannel. (a) Scattered intensity as a function of the frame index (bottom axis) and the recording time (top axis). (b) Histogram of the recorded intensities with a Gaussian fit, showing a relative standard deviation of 1.1%.

8 Mode property of the launching fiber

The delivery fiber used in this work was fabricated in-house. The pattern of the fundamental mode out the fiber output at the wavelength of 532 nm was measured (Fig. S7). The fiber is single-mode with a mode field diameter (MFD) of 8.4 µm. Note that the output beam is approximately Gaussian with a NA of around 0.05.

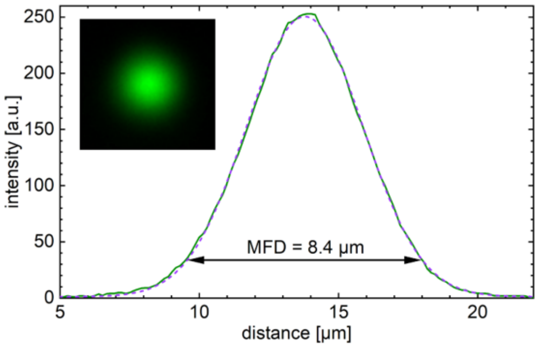

Figure S7: Measured intensity distribution of the output beam of the delivery fiber at the location of the fiber output at the wavelength of 532 nm. The green line refers to the intensity distribution along a selected line in the radial direction, together with a Gaussian fit (purple dashed line). The inset shows the measured distribution.

9 Filling Length

To show the improvement in case the diameter of the microchannel is increased, the filling length of the liquid used in the manuscript ($\eta = 3 \cdot 10^{-3}$ Pa·s, $\gamma = 72.8 \cdot 10^{-3}$ Nm, $\theta = 30°$) [5], [6] is calculated as a function of filling time for two microchannel diameters ($D_{\mathrm{mc}} = 400$nm and $D_{\mathrm{mc}} = 4\mu$m) using the Washburn equation (Fig. S8) [7]. It can clearly be seen that the filling time is significantly shorter for larger channel diameters. For example, a capillary with a hole diameter of $D_{\mathrm{mc}} = 4$ µm can be filled in approximately 8 minutes, while the filling time for a capillary with a diameter of $D_{\mathrm{mc}} = 400$ nm is 78 minutes, an improvement by a factor of about 10.

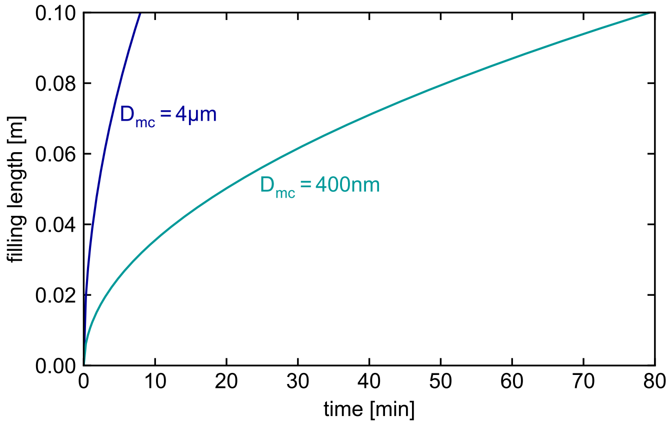

Figure S8: Calculated filling length as function of the filling time.

10 Estimation of concentrations

The minimum, maximum and used NP concentrations are described in the following.

Concentration of the diluted solution: The diluted solution used in the tracking experiments had a NPs concentration of $c_{\mathrm{used}} = 8{\cdot}10^8$ NPs/ml, while the concentration of the original stock solution (nanoComposix Au50_UU) was $4 \cdot 10^{10}$ NPs/ml. The nanospheres solution was diluted to 10% in water and mixed with DMSO to adjust the refractive index of the liquid to that of the silica capillary.

Minimal concentration: The minimum concentration is defined by the fact that at least one NP must be in the field-of-view. Considering the diameter of the microchannel ($D_{\mathrm{mc}} = 4$ µm) and the length of the field of view ($L_{\mathrm{FoV}} = 210$ µm), this gives a volume of $V_{\mathrm{FoV}} = 2.6 \cdot 10^{-9}$ ml and a minimum concentration of $c_{\mathrm{min}} = 1/V_{\mathrm{FoV}} = 3.8 \cdot 10^8$ NPs/ml.

Maximal concentration: The criterion for the estimation of the maximum NP concentration c_{max} is that the trajectories of the NPs must not cross, as this can lead to undesired termination of the tracking algorithm. Here, we assume that only one NP is located within a cross-section of the microchannel (xy-plane), which is achieved experimentally by selecting an appropriate observation area along the fiber. The mentioned condition is emulated in the model by placing the NPs at a constant distance along the fiber axis (Fig. S9(a)) and assuming diffusion only along the z-axis. Due to Brownian motion, the probability distribution for NP occurrence spreads out spatially with time. The corresponding characteristic distance is the diffusion length $L_{\mathrm{dif}} = \sqrt{2D_{\mathrm{dif}}\tau_{\mathrm{m}}}$. If the trajectories of two neighboring NPs should not overlap, no other NP should be within the interval $L = 2 \cdot L_{\mathrm{dif}}$, i.e., volume $V = \pi R_{\mathrm{mc}}^2 L$ (R_{mc}: radius of microchannel). This defines the maximum concentration as $c_{\mathrm{max}} = 1/V = \left(\pi R_{\mathrm{mc}}^2 2 \cdot \sqrt{2D_{\mathrm{dif}}\tau_{\mathrm{m}}}\right)^{-1}$, which decreases with time. The situation for the configuration studied in this work ($R_{\mathrm{mc}} = 2$ µm, $d = 50$ nm) is represented by the cyan curve in Fig. S9(b) (cyan), showing the expected decrease

of c_{max}. For the measurement time used in the experiment ($\tau_{\text{m}} = 65$ s), a maximum NP concentration $c_{\text{max}} = 25 \cdot 10^8$ NPs/ml is obtained (purple vertical dashed-dotted line), which is significantly higher than the used concentration ($c_{\text{used}} = 8 \cdot 10^8$ NPs/ml) and the minimum concentration ($c_{\text{min}} = 3.8 \cdot 10^8$ NPs/ml).

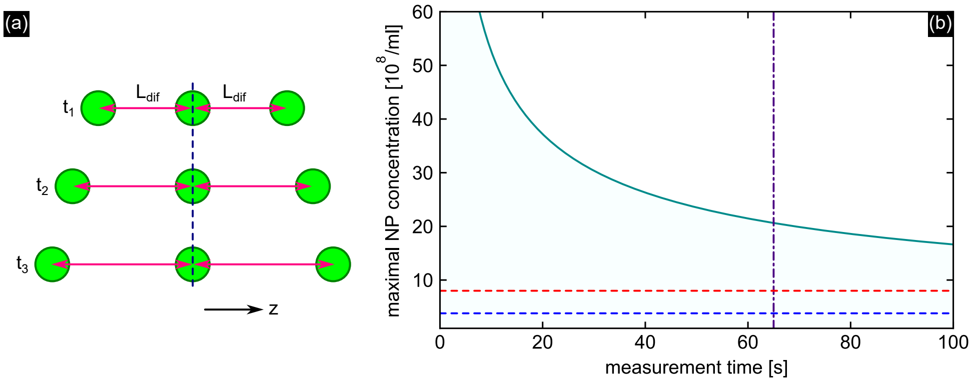

Figure S9: Model for estimating the maximum feasible NP concentration for the used experimental configuration. (a) Sketch of a chain of NPs (green) along the fiber axis (z-axis) at three different points of time ($t_1 < t_2 < t_3$). Note that the distance between NPs is defined by the diffusion length L_{dif} and increases with time. (b) Estimated maximum NP concentration as a function of measurement time (cyan curve). The horizontal dashed red and blue lines indicate the used and minimum concentration ($c_{\text{used}} = 8 \cdot 10^8$ NPs/ml, $c_{\text{min}} = 3.8 \cdot 10^8$ NPs/ml). The vertical purple dashed line refers to the measurement time of the experiments reported here ($\tau_{\text{m}} = 65$ s).

11 Estimation of critical intensities

In the following it is estimated from which power and intensity values the result of the MSD-based diameter determination changes and whether these values are reached with the exponents discussed here, i.e., whether heating of the particles must be taken into account.

A. Localized surface plasmon resonance: From a nanoparticle scattering perspective, the key parameter in our experiments is the scattering cross-section (σ_{scat}), which is plotted as a function of wavelength in Fig. S10. The data show that within the spectral range of interest (the visible spectral domain, 500 nm< λ_0 <700 nm), the variation of σ_{scat} below one order of magnitude. Therefore, the influence of plasmonic effects on the optical properties (i.e., the variation of the scattering intensity) can be considered minimal in case the operation wavelength is changed.

B. Impact of plasmonic absorption: The metallic composition of the NPs and their inherent plasmonic properties potentially affect the experiments reported here through the following two mechanisms:

B.1. Locally Induced Heat: Due to the significant absorption properties of plasmonic NPs, there is a critical intensity at which the induced heat changes the local temperature and consequently reduces the viscosity of the surrounding liquid. This phenomenon has been extensively studied in one of our previous studies [10], which investigated the limitations of the FaNTA method using a single anti-resonant element fiber. In this study, we analyzed the diffusion behavior of NPs of similar dimensions (gold, 50 nm in diameter, water) within microstructured optical fibers containing one single optofluidic microchannel of much larger diameter (diameter 17 µm). Following this analysis, the critical intensity related to induced heat $I_{\text{crit}}^{\text{heat}}$ — the threshold at which the heat generated locally affects the experimental results — was estimated using the outlined procedure:

1. *Critical output power:* Figure S-5 in the Supplementary Information of reference

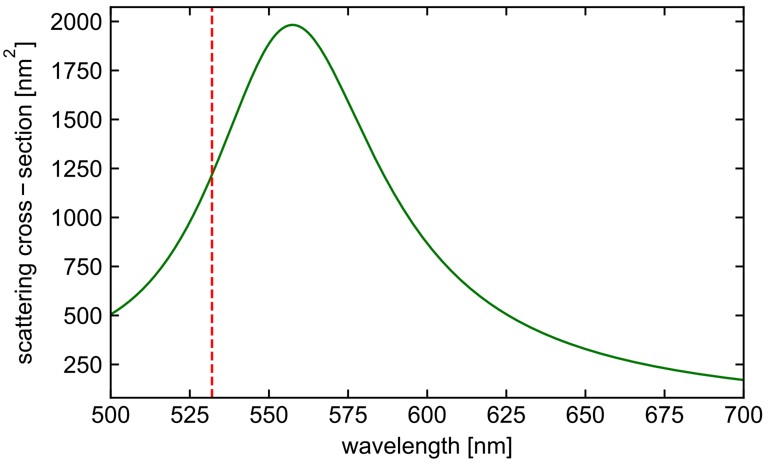

Figure S10: Scattering cross-section σ_{scat} of a gold NP (diameter d=50 nm) in a medium that has the refractive index of silica ($n_{\text{background}} = n_{\text{silica}}$). The vertical dashed red line indicates the operation wavelength used in the manuscript.

[10] shows the determined median diameter of an ensemble of 50 nm gold NPs as a function of laser output power. This plot illustrates the critical output power at which thermal effects begin to affect the measurements, which was found here to be $P_{\text{crit}}^{\text{output}} = 4.3$ mW.

2. *Critical power at the measurement site:* As the NTA experiments were performed close to the input of the anti-resonant fiber, the power at the observation site was calculated taking into account the modal attenuation. This analysis resulted in a critical NTA power of $P_{\text{crit}} = 9$ mW.

3. *Critical intensity:* The critical intensity at which temperature affects MSD calculations is determined by the equation

$$P_{\text{crit}} = \int I(x,y)dA = I_{\text{crit}}^{\text{heat}} \cdot \int f(x,y)dA \tag{4}$$

with the maximum intensity I_0 and the normalized modal distribution $f(x,y) = S_z(x,y)/S_z(0,0)$, which is defined by the longitudinal Poynting vector $S_z(x,y)$ (note that $f(0,0) = 1$). The integration extends infinitely over the xy-plane. The Poynting vector distribution of the fundamental mode of the anti-resonant fiber was calculated using the 'nanobore model' previously used to calculate fields within a step-index fiber with a central nanochannel [3]. The critical intensity for 50 nm NPs diffusing in water then calculated as $I_{\text{crit}}^{\text{heat}} = 0.07$ mW/µm^2.

To calculate the intensity in the nanochannel, an analogous approach can be employed which effectively integrates the intensity distribution shown in Fig. 2(a) at $z = 1$ mm and considers the power at the output of the delivery fiber ($P_{\text{output}} = 17$ mW), leading to an intensity of $I_{\text{mix}} = 0.0163$ mW/µm^2 for the experiments operating in the DMSO/water environment. Correcting this value with the different thermal conductivities of water ($k_{\text{water}} = 0.6$ W/mK [8]) and the DMSO/water mixture ($k_{\text{mix}} = 0.2$ W/mK [9]) gives $I_{\text{correct}} = I_{\text{mix}} \cdot k_{\text{mix}}/k_{\text{water}} \approx 3 \cdot I_{\text{mix}} \approx 0.049$ mW/µm^2. The resulting value is well below the previously determined critical intensity, indicating that the heating can be neglected.

B.2. Photon pressure: A comparable procedure can be employed to assess the impact of photon pressure on the experiments, as illustrated in Fig. S-4 in the Supplementary Information of Ref. [10]. This plot indicates a level of critical output power that is comparable to that of the previous section, resulting in a critical photon-pressure-related intensity of $I_{\text{crit}}^{\text{press}} = 0.07$ mW/µm^2. This intensity is once again higher than that employed

in the present study. Consequently, the impact of photon pressure is not considered in this study.

C. Transparent nanoparticles (NPs): The employment of transparent NPs is possible as the measurement principle is independent of plasmonic effects. A number of studies using NTA within optical fibers have been reported, including the characterization of (i) dielectric nanoparticle ensembles in single-element anti-resonant fibers [11], as well (ii) lambda phages in hollow-core fibers [12]. It is important to note that the scattering cross-section of dielectric nanoparticles is considerably smaller than that of their plasmonic counterparts. Consequently, transparent NPs are typically larger in NTA experiments to provide sufficient scattering signal.

12 Mathematical symbols used

Table 1: List of symbols used in this work.

Symbol	Unit	Explanation
z_{a}	m	distance between delivery fiber and capillary
Δz	m	distance between capillary input and measurement area
R_{mc}	m	radius of microchannel
D_{mc}	m	diameter of microchannel
L_{FoV}	m	length of field-of-view
V_{FoV}	m	volume of field-of-view
c_{used}	$1/\mathrm{m}^3$	used nanoparticle concentration
c_{min}	$1/\mathrm{m}^3$	minimum nanoparticle concentration
c_{max}	$1/\mathrm{m}^3$	maximum nanoparticle concentration
ω_0	m	waist of Gaussian beam
d	m	physical diameter of nanoparticle
d_{h}	m	hydrodynamic diameter
D_{dif}	m^2/s	diffusion coefficient
L_{dif}	m	diffusion length
$\Delta I(L_{\mathrm{dif}})$	1	intensity decrease over the diffusion length
N	1	number of frames of recorded trajectory
τ_{m}	s	total measurement time
ν	Hz	frame rate
τ_{e}	s	exposure time
P_{output}	mW	output power
I	1	scattering intensity
$\bar{I}$	1	average scattering intensity
σ_{I}	1	measured standard deviation of scattering intensity
δI	1	relative standard deviation of scattering intensity

References

[1] M. Born and E. Wolf, *Principles of optics: electromagnetic theory of propagation, interference and diffraction of light*, 6th ed., Elsevier, 2013.

[2] G. M. Hale and M. R. Querry, "Optical constants of water in the 200-nm to 200-µm wavelength region," *Appl. Opt.*, vol. 12, no. 3, pp. 555–563, 1973.

[3] F. Gui, et al., "Light strands: exploring flat-field modes in optofluidic fibers for tracking single nano-objects," *Optica*, vol. 10, no. 6, pp. 717–724, 2023.

[4] K. A. Akmarov, S. N. Lapshov, A. S. Sherstobitova and A. D. Yas'kov, "Optical properties of aqueous solutions of dimethyl sulfoxide and application of refractometry for monitoring their composition," *J. Appl. Spectrosc.*, vol. 80, no. 4, pp. 610–614, 2023.

[5] E. W. Lemmon, "Thermophysical properties of fluid systems," NIST chemistry WebBook, 2010.

[6] S. Iglauer, A. Salamah, M. Sarmadivaleh, K. Liu, and C. Phan, "Contamination of silica surfaces: Impact on water–co2–quartz and glass contact angle measurements," *Int. J. Greenhouse Gas Control*, vol. 22, pp. 325–328, 2014.

[7] E. W. Washburn, "The dynamics of capillary flow," *Phys. Rev.* vol. 17, no. 3, p. 273, 1921.

[8] Z. Qin and J.C. Bischof, "Thermophysical and biological responses of gold nanoparticle laser heating," *Chem. Soc. Rev.*, vol. 41, no. 3, pp. 1191–1217, 2012.

[9] C. Nieto-Draghi, J. B. Avalos and B. Rousseau, "Transport properties of dimethyl sulfoxide aqueous solutions," *J. Chem. Phys.*, vol. 119, no. 9, pp. 4782–4789, 2003.

[10] T. Wieduwilt, R. Förster, M. Nissen, J. Kobelke, and M. A. Schmidt, "Characterization of diffusing sub-10 nm nano-objects using single anti-resonant element optical fibers," *Nat. Commun.*, vol. 14, no. 1, p. 3247, 2023.

[11] M. Nissen, et al., "Nanoparticle tracking in single-antiresonant-element fiber for high-precision size distribution analysis of mono-and polydisperse samples," *Small*, vol. 18, no. 38, p. 2202024, 2022.

[12] R. Foerster, et al., "Tracking and analyzing the Brownian motion of nano-objects inside hollow core fibers," *ACS Sens.*, vol. 5, no. 3, pp. 879–886, 2020.
